# Porcine Reproductive and Respiratory Syndrome Virus (PRRSV)-Induced Reactive Oxygen Species Inhibit Phagocytosis in Alveolar Macrophages

**DOI:** 10.3390/ijms27135800

**Published:** 2026-06-26

**Authors:** Yuhao Xia, Yihan Li, Junwei Wang, Mengting Zhang, Jiahui Li, Zhuosong Yang, Shijie Zhao, Yanan Wu, Jing Chen, Yina Zhang, Honglian Dai, Mengxiang Wang

**Affiliations:** 1 State Key Laboratory of Advanced Technology for Materials Synthesis and Processing, Wuhan University of Technology, Wuhan 430070, China; 2 College of Veterinary Medicine, Henan Agricultural University, Zhengzhou 450046, China; 15617356215@163.com (Y.L.); zmt6688999@163.com (M.Z.); z861969939@163.com (J.L.); zsj1002935527@163.com (S.Z.); wlyananjiayou@yeah.net (Y.W.); yinazhang2020@henau.edu.cn (Y.Z.); 3 College of Life Science, Henan Agricultural University, Zhengzhou 450046, China; wangjunwei030600@163.com (J.W.); yzs15937216927@gmail.com (Z.Y.); chenjing@henau.edu.cn (J.C.)

**Keywords:** PRRSV, phagocytosis, phagosome, PRV-pAb complexes, ROS

## Abstract

Porcine reproductive and respiratory syndrome (PRRS) is an immunosuppressive disease caused by PRRS virus (PRRSV). PRRSV infection not only compromises the host immune defenses, but also predisposes the host to secondary infections by other pathogens, of which PRV is one of the common secondary infection pathogens. Porcine alveolar macrophages (PAMs) are the primary target cells of PRRSV, and their phagocytic function is critical for immune defense, homeostasis maintenance, and disease regulation. However, PRRSV disrupts PAMs phagocytosis, impairing the host’s ability to combat infection. This study used PRV-pAb complexes as phagocytic indicators, investigated the effect of PRRSV infection on PAMs phagocytosis and its underlying molecular mechanisms. We found that PRRSV infection interfered with phagosome maturation—a process regulated by Rab7 and other regulators, thereby blocking phagocytic degradation and significantly suppressing PAMs phagocytic activity. Further analysis revealed that reactive oxygen species (ROS) play a key role in this process. Elevated ROS levels damaged lysosomal membrane integrity, ultimately inhibiting phagosome-lysosome fusion. Notably, phagocytosis of PRRSV-infected PAMs was partially restored with N-acetylcysteine (NAC) by reducing ROS levels. These findings offer novel insights into PRRSV-induced immunosuppression and secondary infections while providing a theoretical foundation for developing more effective PRRSV prevention and control strategies.

## 1. Introduction

Porcine Reproductive and Respiratory syndrome (PRRS), also known as porcine blue ear disease (PBED), is a globally significant infectious disease caused by the Porcine Reproductive and Respiratory Syndrome virus (PRRSV). First reported in the United States in 1987, PRRS has since spread worldwide, causing substantial economic losses to the swine industry [1,2,3]. Clinically, PRRS is characterized by respiratory disorders in pigs of all ages and reproductive failure in gestating sows. PRRSV is a single-stranded, positive-sense RNA virus with a genome encoding 11 known open reading frames (*ORFs*) [4]. The replicase genes (*ORFs 1a* and *1b*) encode two large nonstructural polyproteins (pp1a and pp1ab), which are cleaved into at least 14 nonstructural proteins (NSPs). The remaining *ORFs* (*ORF2-7*) encode four glycosylated membrane proteins (GP2a, GP3, GP4, and GP5), three non-glycosylated membrane proteins (E, ORF5a, and M), and the nucleocapsid protein (N) [1,5,6,7]. Due to the high genomic variability, PRRSV frequently evolves into new variants. In 2012, a NADC30-like recombinant strain emerged and has since become the dominant circulating strain, leading to increased infection rates and complicating disease control [8,9,10]. Furthermore, recent studies indicate that inter-strain recombination has enhanced PRRSV’s genetic diversity, posing additional challenges for diagnosis, prevention and disease management [11,12,13,14].

Upon invading the host organism, PRRSV primarily targets the lungs, causing lung damage, with porcine alveolar macrophages (PAMs) serving as the primary cells of infection [15,16,17]. Given that PAMs play a pivotal role in the innate defense mechanisms of the lungs, PRRSV disrupts the respiratory immune system of pigs during the early stages of infection. Simultaneously, the lung damage caused by PRRSV weakens the overall immune response of the pig, thereby predisposing them to secondary infections [18,19]. For example, the replication pathways of PRV (porcine pseudorabies virus) in pigs are primarily divided into primary replication in the upper respiratory tract, lymphatic and hematogenous spread, neural latency, and secondary replication in target organs. Although PRV can infect various porcine cell types, studies indicate that it exhibits a strong tropism for neural tissue, while infection of immune cells such as PAMs is virtually absent [20,21]. Literature reports indicate that porcine pseudorabies virus (PRV), circovirus type 2 (PCV2) and swine influenza virus (SIV) are frequently associated with secondary or synergistically infection following PRRSV infection in pigs [22,23,24,25]. Piglets infected with PRRSV exhibit a morbidity rate approaching 100%, with a mortality rate exceeding 50%. When co-infected with viruses such as PCV2, the mortality rate among piglets can escalate to nearly 60% [26,27,28]. The underlying factor driving these phenomena is PRRSV-induced immunosuppression, characterized by macrophage destruction and T-cell dysfunction. This, coupled with the virus’s high susceptibility to genomic mutation, renders PRRSV prevention and control exceptionally challenging [29,30]. Therefore, research focusing on the functional suppression of PAMs by PRRSV is of paramount importance.

Phagocytosis in mammalian macrophages represents an important physiological process and serves as a cornerstone of the body’s immune defense [31]. It acts as the first line of defense against pathogen and is a key step in initiating adaptive immunity, playing a vital role in immune regulation and inflammatory homeostasis [32]. During this process, cells internalize antigens (e.g., bacteria, viruses, immune complexes, or other large particulate matter) by engulfing them at the cell surface via cell membrane-bound receptors, forming phagosomes [33]. The newly formed phagosome then fuses with early endosomes in a membrane fusion event regulated by the small GTPase Rab5, which recruits the molecule EEA1 (early endosomal antigen 1) to facilitate this fusion [34,35]. Concurrently, vesicles termed circulating endosomes are expelled from the phagosome. Subsequently, EEA1, acting as a bridge between early endosomes and phagosomes, promotes the recruitment of late endosomal effector proteins, such as Rab7 [36,37]. As phagosome maturation progresses, Rab5 is lost, and Rab7 appears on the membrane [36]. Rab7 then mediates the fusion of phagosomes with late endosomes [37]. During this fusion with late endosomes, V-ATPase molecules accumulate on the phagosome membrane, responsible for acidifying the phagosomal interior (pH 5.5–6.0) by translocating protons (H+) into the phagosomal lumen [38]. As the late endosome approaches the lysosome, membrane contact sites (MCSs) are formed, triggering the accumulation of lipid signaling molecules such as phosphatidylinositol 3,5-bisphosphate (PI(3,5)P_2_). This lipid signal specifically activates the transient receptor potential mucolipin 1 (TRPML1) channel embedded in the lysosomal membrane. Upon TRPML1 channel opening, the high concentration of calcium ions (Ca^2+^) sequestered within the lysosome is released into the cytoplasm, generating a localized microdomain Ca^2+^ signal. This spatially restricted calcium flux then activates calcium-sensitive effector proteins, including calmodulin (CaM), which in turn promotes the assembly of soluble N-ethylmaleimide-sensitive factor attachment protein receptor (SNARE) complexes—such as the VAMP7-syntaxin 7 pairing—to drive membrane fusion between the late endosome and lysosome [39]. In the final stage of phagosome maturation, the phagosome fuses with the lysosome to form the phagolysosome. Phagolysosomes exhibit marked acidity due to the accumulation of V-ATPase on their membranes [40]. Ultimately, phagolysosomes can digest the antigens to generate immunogenic peptides or degrade them directly.

When phagocytosis is impaired, pathogens can evade clearance and proliferate. Previous studies have shown that PRRSV can significantly inhibit the phagocytic capacity of PAMs, leading to persistent viral infection and lung tissue damage, triggering interstitial pneumonitis and lymphadenopathy, resulting in immunosuppression. This immunosuppression interferes with and undermines the pigs’ resistance to other pathogens, thereby causing mixed or secondary infections with multiple pathogens [1,25]. While existing research on PRRSV has mainly focused on epidemiology and viral structure, a detailed description of how PRRSV affects PAM phagocytosis remains lacking, which is a critical issue.

In this study, we investigated the impact of PRRSV infection on phagocytosis in PAMs and its underlying molecular mechanisms. Utilizing the PRV-pAb complex as a measure of phagocytic activity, we determined that PRRSV infection significantly inhibits phagocytosis in PAMs and disrupts lysosomal homeostasis, thereby impeding phagosome-lysosome fusion and subsequent phagosomal degradation. Notably, reactive oxygen species (ROS) played a pivotal role in this process and were the primary causative factor for the observed outcomes. These findings offer new insights into the phenomenon of PRRSV-induced immunosuppression and secondary infection, providing a theoretical foundation for the development of novel prevention and treatment strategies against PRRSV infection.

## 2. Results

### 2.1. PRRSV Significantly Decreased the Phagocytic Activity of PAMs

**Figure 1:**
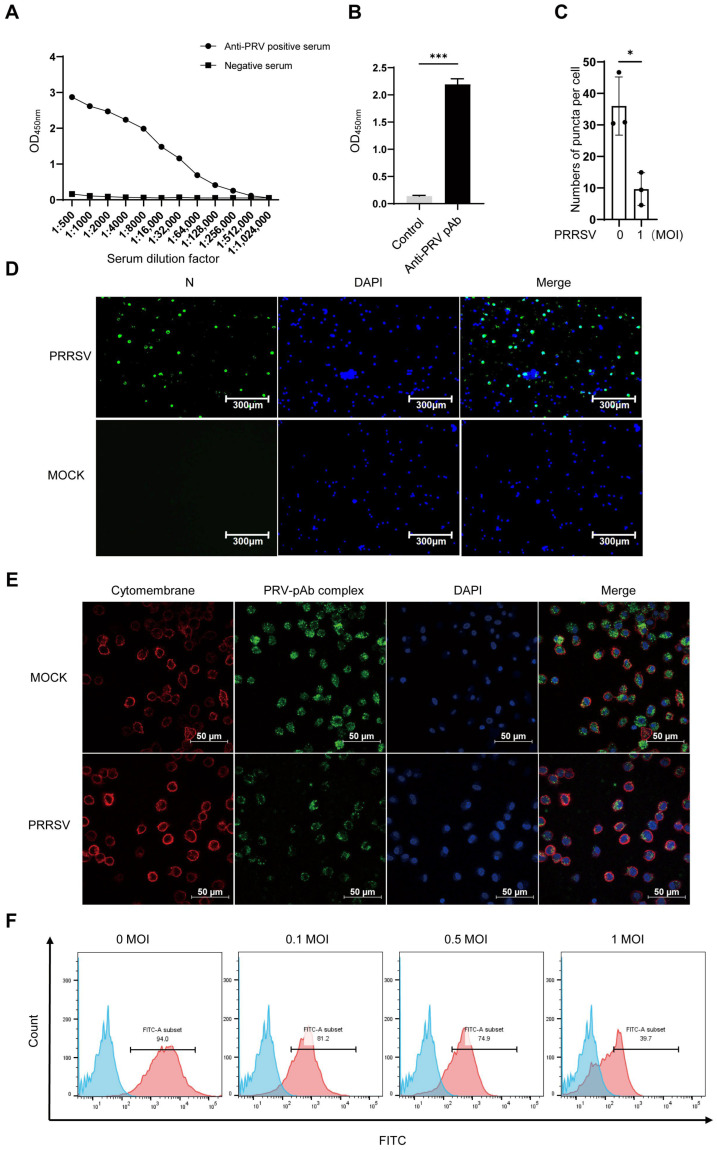
PRRSV inhibits the phagocytic activity of PAMs. (**A**), PRV was immobilized on ELISA plates, and different dilutions of anti-PRV serum were employed as primary antibodies to determine the antibody titer. (**B**), PRV was coated onto ELISA plates. Purified anti-PRV antibody was used as the primary antibody, and the reactivity of the purified antibody with PRV was measured. (**C**), Quantification of the number of PRV-pAb puncta per cell, derived from experiments in (**E**). The bars represent the mean ± SEM of the number of individual cells across three independent experiments. (**D**), Confocal microscopy images of PAMs infected with PRRSV after incubation with anti-PRRSV N protein antibody. Cell nuclei were stained with DAPI. Scale bar: 300 μm. (**E**), Confocal microscopy images of PRRSV-infected PAMs incubated with PRV-FITC-pAb. Nuclei were stained with DAPI, and cell membranes were stained with iFluor 594 wheat germ agglutinin (WGA) affix. Scale bar: 50 μm. (**F**), Cells harvested from the experiment in (**D**) were subjected to flow cytometry assays. The blue curve represents untreated cells, while the red curve represents cells incubated with a complex formed by the binding of FITC-labeled anti-PRV polyclonal antibodies to PRV. The scale represents the percentage of cells that have phagocytosed the complex. In (**B**,**C**), * *p* < 0.05, *** *p* < 0.001, as determined by the unpaired Student’s *t* test.

We utilized the PRV-antigen–antibody complex as a metric to assess the phagocytic activity of PAMs. Normally, PRV does not infect PAMs. Instead, PRV binds to anti-PRV antibodies, forming antigen–antibody complexes. These complexes are then engulfed by PAMs, generating phagocytic vesicles. These vesicles subsequently fuse with lysosomes, forming phagolysomes, which are ultimately degraded. We found that the titer of the anti-PRV serum could exceed 1:256,000 (Figure 1A). This high-titer serum could be used to bind with PRV, thereby forming the antigen–antibody complexes. After purifying the PRV antiserum, we coated an ELISA plate with inactivated PRV antigen and used the purified polyclonal antibody as the primary antibody. The results showed that the optical density (OD) value exceeded 2, which was significantly higher than that of the negative control, indicating that the purified polyclonal antibody exhibited robust reactivity against PRV (Figure 1B). PAMs were infected with PRRSV for 12 h, followed by incubation with anti-PRRSV N protein antibody as the primary antibody. The results showed distinct fluorescence signals in PRRSV-infected PAMs, whereas no fluorescence was detected in the control group, confirming that the PRRSV stock preserved in our laboratory can effectively infect PAMs (Figure 1D).

The purified PRV antibody was then conjugated with FITC. The complex formed by the binding of the FITC-labeled anti-PRV polyclonal antibody to PRV was added to the PAMs that had been subjected to specific treatments. The PAMs were collected 1.5 h later for observation. Confocal fluorescence microscopy revealed that the number of PRV-antibody complexes phagocytosed by PRRSV-infected PAMs was significantly lower compared to that in normal, uninfected cells (Figure 1C,E). Additionally, we measured the intracellular FITC content using flow cytometry. In the control group without PRRSV infection, approximately 94% of cells showed phagocytosis of the PRV-pAb complex. whereas upon infection with PRRSV at 1 MOI, this proportion decreased to 39.7%. The results indicated that the reduction phagocytic activity in response to PRRSV inoculation occurred in a dose-dependent manner (Figure 1F). Collectively, these findings suggest that PRRSV significantly diminishes the phagocytic activity of PAMs, leading to a decrease in the overall phagocytic capacity of these cells.

### 2.2. PRRSV Blocks Phagocytic Channels in PAMs

**Figure 2:**
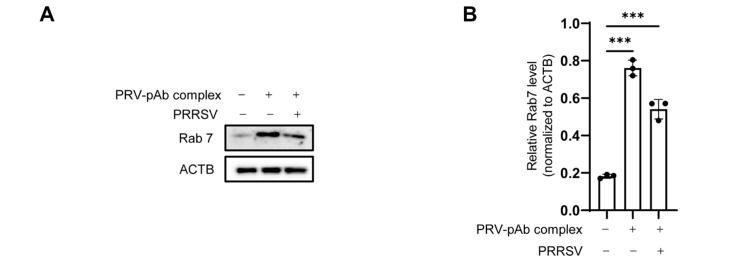

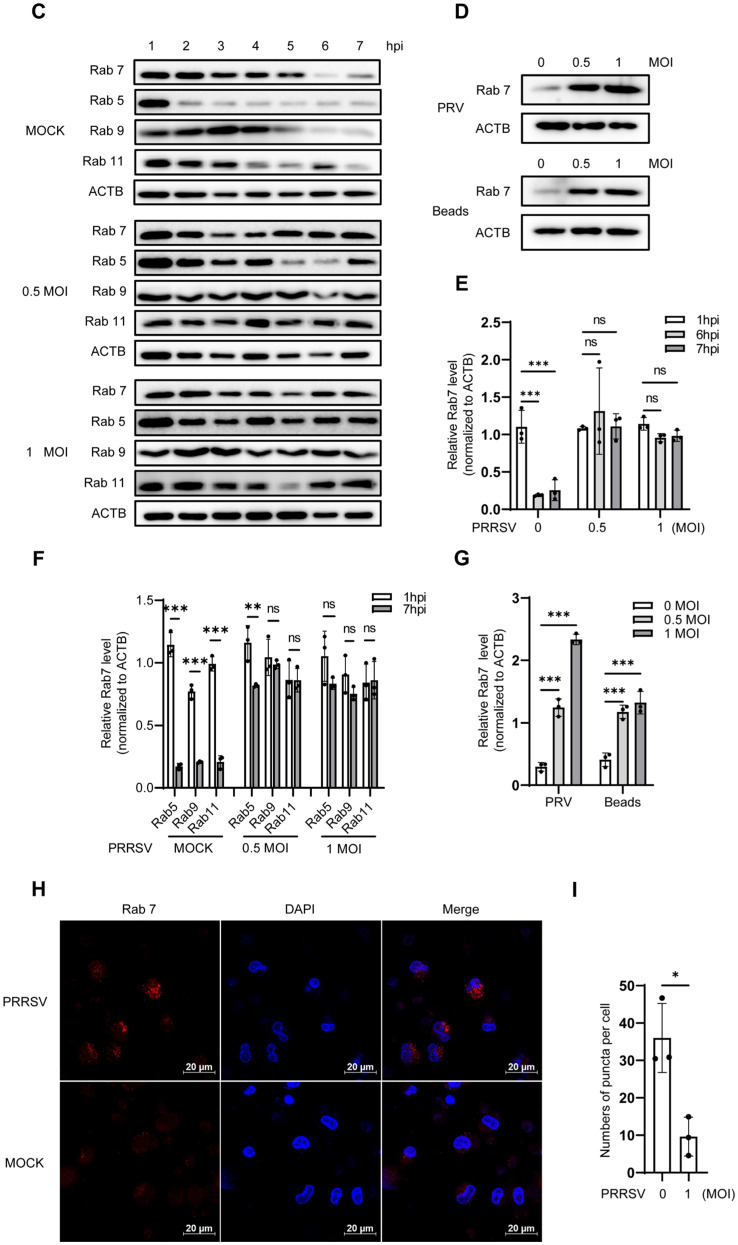
PRRSV induces blockage of phagocytic channels in PAMs. (**A**), Immunoblotting analysis was performed on PAMs treated with 1 MOI PRRSV and/or PRV-pAb complex. Cells treated with PRRSV were harvested after 24 h, while those incubated with the complex were collected after 1 h. (**B**), Quantification of Rab7 levels, normalized to ACTB, derived from experiments conducted as described in (**A**). The Rab7: ACTB ratio for the control group was arbitrarily assigned a value of 1. Bars represent the mean ± SEM from three independent experiments. (**C**), PAMs were exposed to PRRSV at the indicated viral loads for 24 h, followed by incubation with PRV-pAb complexes. Samples were collected at specified intervals and subjected to immunoblotting analysis. (**D**), PAMs were treated with PRRSV at the indicated viral loads for 24 h, then incubated with either PRV or Protonex^TM^ 600 Red-latex beads conjugate (Beads) for 7 h, prior to immunoblotting analysis. (**E**), Quantification of Rab7 levels, normalized to ACTB, from experiments conducted as in (**C**). The Rab7: ACTB ratio for the control group was set at 1. Bars represent the mean ± SEM from three independent experiments. (**F**), Quantification of Rab5, Rab9, and Rab11 levels, normalized to ACTB, derived from experiments conducted as described in (**C**). The ratio of each indicated protein to ACTB for the control group was arbitrarily set at 1. Bars represent the mean ± SEM from three independent experiments. (**G**), Quantification of Rab7 levels, normalized to ACTB, from experiments as in (**D**). The Rab7: ACTB ratio for the control group was set at 1. Bars represent the mean ± SEM from three independent experiments. (**H**), Confocal microscopy images of PRRSV-treated PAMs immunostained for endogenous Rab7. Nuclei were stained with DAPI. Scale bar: 20 μm. (**I**), Quantification of the number of Rab7 puncta per cell from experiments depicted in (**H**). Bars represent the mean ± SEM of the number per cell from three independent experiments. In (**B**,**E**,**G**), statistical significance was determined using one-way analysis of variance (ANOVA) with Dunnett’s multiple comparisons test (ns: no significant difference, *** *p* < 0.001). In (**F**), statistical significance was determined using one-way ANOVA with Tukey’s multiple comparisons test. In (**I**), statistical significance was assessed using the unpaired Student’s *t* test (ns: no significant difference, * *p* < 0.05, ** *p* < 0.01, *** *p* < 0.001).

To elucidate the effect of PRRSV on phagocytosis, we initially analyzed the expression level of Rab7, a small G protein in the Rab family, in PRRSV-treated PAMs. Rab7 plays a pivotal role in the maturation of lysosomes and phagosomes and is a key regulator in the fusion processes of phagocytosis/autophagy vesicles and lysosome. The treated PAMs were incubated with PRV-pAb complexes for 1 h, followed by immunoblotting for Rab7. No distinct bands were observed in the control group; however, Rab7 protein levels increased significantly following treatment with the PRV-pAb complex. The results demonstrated that the complexes were efficiently phagocytosed by PAMs, leading to the formation of phagocytic vesicles and subsequent late endosomes (Figure 2A,B). Samples were then collected at hourly intervals after the addition of the complex.

In normal cells, a gradual decrease in Rab7 protein levels was observed, indicating that PAMs were progressively completing degradation of the PRV-pAb complexes, endosomal Rab7 levels returned to baseline by 6 h post-phagocytosis. In contrast, in PRRSV-infected cells, Rab7 levels remained elevated at 6–7 h, showing no significant difference from levels detected at 1 h post-phagocytosis (Figure 2C,E). To further validate this discrepancy between control and infected groups at 7 h, we performed immunofluorescence assay (IFA) on samples collected 7 h after PAMs phagocytosed the PRV-pAb complex. Quantitative analysis revealed that Rab7 fluorescence intensity in mock-infected cells was significantly lower than in PRRSV-infected cells, further confirming that even 7 h post-complex addition, late endosomes remained more abundant in PRRSV-infected PAMs compared to control cells (Figure 2H,I). Subsequently, to investigate whether PAM produced the same results with PRV and Protonex^TM^ 600 Red-latex beads conjugate (Beads) before and after PRRSV infection, we attempted to co-incubate PAMs with PRV and Protonex^TM^ 600 Red-latex beads conjugate (Beads). We found that PRRSV-infected cells contained higher levels of Rab7 compared to normal cells (Figure 2D,G), indicating that the effect of PRRSV on PAM phagocytosis is not limited to the PRV-pAb complex. Taken together, the results suggest that both immune complexes, viruses, and large particulate matter can induce phagocytosis and stimulate the production of late phagocytic vesicles. However, PRRSV-infected PAMs exhibit a reduced capacity to degrade these substances.

To determine whether the effect of PRRSV on the phagocytosis in PAMs is confined to the accumulation of late phagocytic vesicles, we assayed the early endosomal marker Rab5, the recycling endosomal marker Rab11, and another marker of late endosomes, Rab9. In uninfected PAMs, the levels of these markers exhibited a temporal decline following complex addition, with Rab5 and Rab9 levels stabilizing after 1 h and 5 h, respectively. Subsequently, the recycling endosomal marker Rab11 also decreased after 6 h. This demonstrates that the processing of the pathogenic complexes by normal cells may be completed within 6 h, culminating in the recycling of various intracellular substances. In contrast, in PAMs infected with PRRSV at an MOI of 0.5, Rab5 levels only began to gradually decrease after 5 h post-phagocytosis. Notably, in the 1 MOI PRRSV group, no significant differences were observed in the overall band intensities of Rab5, Rab9, and Rab11 throughout the experimental period, suggesting that restoration to baseline levels may require a longer duration. Collectively, these results indicate that phagosome contents did not undergo significant alterations during the entire process, whether in the early or late stages following phagocytosis (Figure 2C,F). These findings suggest that normal cells efficiently complete the sorting, degradation, and recycling of pathogen complexes within 6 h by precisely regulating Rab protein activity and endosome maturation processes. However, these processes are impeded following PRRSV infection.

### 2.3. PRRSV Inhibits the Binding of Phagocytic Vesicles to Lysosomes, Thereby Inhibiting Their Degradation

**Figure 3:**
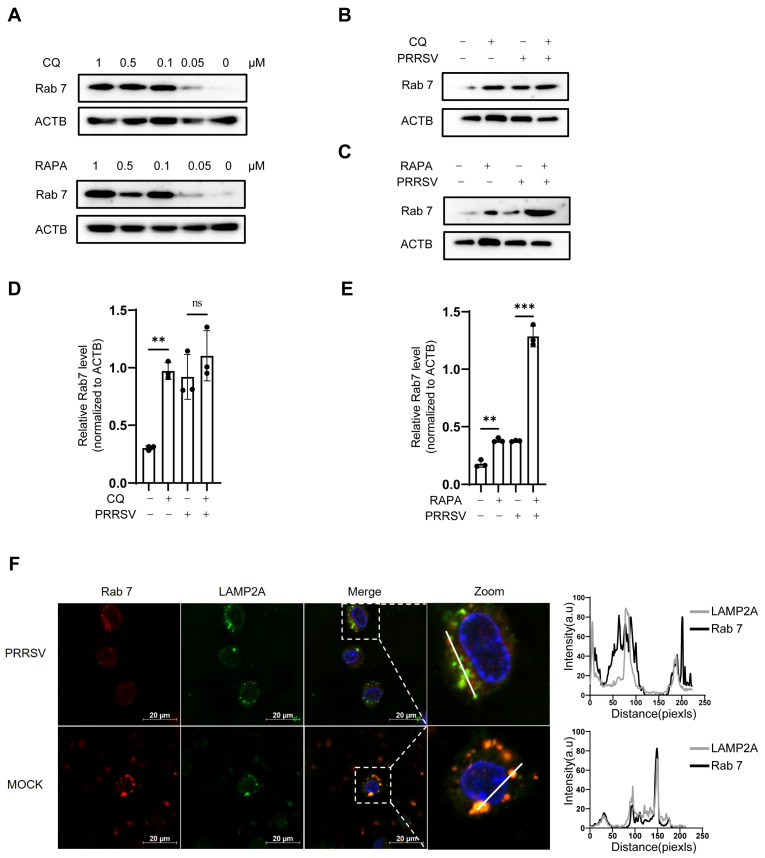
PRRSV inhibits the binding of phagocytic vesicles to lysosomes, thereby impeding the degradation of these vesicles. (**A**), For the CQ treatment, PAMs were incubated with the PRV-pAb complex while the indicated concentration of CQ was added. Samples were collected after 7 h for immunoblot analysis. For the RAPA treatment, PAMs were exposed to RAPA at the indicated concentrations for 7 h, and subsequently analyzed by immunoblotting. (**B**), Immunoblots depicting the effects on PAMs treated with 1 MOI of PRRSV and/or CQ. PAMs were incubated with the PRV-pAb complex for 7 h. Additionally, cells treated with PRRSV for 24 h and/or CQ for 7 h were collected. (**C**), Immunoblots showing the outcomes for PAMs treated with 1 MOI of PRRSV and/or RAPA. PAMs were incubated with the PRV-pAb complex for 7 h. Moreover, cells treated with PRRSV for 24 h and/or RAPA for 7 h were harvested. (**D**), Quantification of Rab7 levels (normalized to ACTB) from experiments similar to those in (**B**). The ratio of Rab7: ACTB for the control group was arbitrarily set at 1. Bars represent the mean ± SEM from three independent experiments. (**E**), Quantification of Rab7 levels (normalized to ACTB) derived from experiments as those in (**C**). The ratio of Rab7: ACTB for the control group was arbitrarily set at 1. Bars represent the mean ± SEM from three independent experiments. (**F**), PAMs were treated with 1 MOI of PRRSV for 24 h, followed by incubation with the PRV-pAb complex for 7 h. Subsequently, the cells were immunostained with antibodies against Rab7 and LAMP2A. Nuclei were stained with DAPI. Scale bar: 30 μm. The pixel intensity distribution and correlation in different fluorescence channels along the white line were analyzed using ImageJ (v 1.4.3.67). In (**D**,**E**), ns: no significant difference, ** *p* < 0.01, *** *p* < 0.001, determined by one-way ANOVA with Tukey’s multiple comparisons test.

To determine how PRRSV affects phagocytosis in PAMs, we initially sought to determine whether the elevated Rab7 levels in PRRSV-treated cells were attributable to increased synthesis or decreased degradation. The mTOR inhibitor rapamycin is known to augment endosomal content by activating the autophagy pathway. We administered different doses of rapamycin to PAMs and observed a dose-dependent increase in Rab7 levels (Figure 3A). Chloroquine (CQ) can prevent endosome degradation by modulating lysosomal acidification, thus inhibiting lysosomal fusion with various endosomes. We treated PAMs, which had been incubated with PRV-antibody complexes, with CQ. The results showed that Rab7 degradation was inhibited (Figure 3A). Building on these findings, we infected the cells with PRRSV, followed by the addition of PRV-pAb complexes and treatment with different drugs. The results demonstrated that CQ treatment resulted in equally high levels of Rab7 in both control and PRRSV-treated cells, with an approximate 3.19-fold increase compared to untreated control cells (Figure 3B,D). In contrast, rapamycin elevated Rab7 levels in both PRRSV-treated and control cells, with the difference in Rab7 levels between the two conditions persisting (Figure 3C,E). These experiments suggest that PRRSV treatment does not increase Rab7 levels due to enhanced phagocytic vesicle formation but rather blocks phagocytic vesicle degradation (i.e., phagocytic flux). Subsequently, we conducted co-localization studies of phagocytic vesicles with lysosomes. The results revealed that in normal cells, phagocytic vesicles could co-localize with lysosomes following the addition of PRV-pAb complexes, and phagocytic vesicles and lysosomes could fuse normally to form a phagolysosome. However, this co-localization was significantly diminished after infection with PRRSV (Figure 3F). This finding indicates that PRRSV may inhibit the degradation of phagocytosis vesicles by impeding their binding to lysosomes, thereby preventing their degradation.

### 2.4. PRRSV Induces Lysosomal Membrane Permeabilization

**Figure 4:**
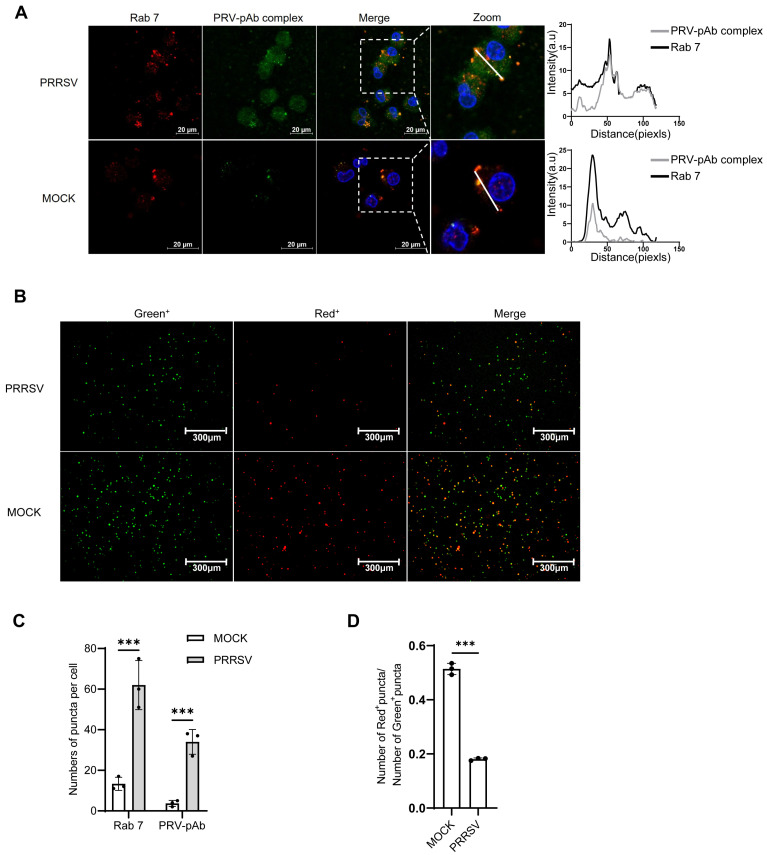
PRRSV induces lysosomal membrane permeabilization. (**A**), PAMs were treated with 1 MOI of PRRSV for 24 h and then incubated with FITC-PRV-pAb complex for 7 h. Subsequently, the cells were immunostained with antibodies against Rab7. Nuclei were stained with DAPI. Scale bar: 20 μm. The pixel intensity distribution and correlation in different fluorescence channels along the white line were analyzed using ImageJ (v 1.4.3.67). (**B**), PAMs were treated with 1 MOI of PRRSV for 24 h, after which Protonex™ 600 Red-latex beads conjugate was added for 4 h. CytoTrace™ Green working solution was then added for 0.5 h, and the cells were observed using Zeiss laser scanning confocal microscope (Zeiss, Oberkochen, Germany). Scale bar: 300 μm. (**C**), Quantification of the number of Rab7 puncta per cell derived from experiments in (**A**). Bars represent the mean ± SEM of the puncta count per cell from three independent experiments. (**D**), he ratio of the number of green-red-positive puncta to red-positive puncta within the same field of view was determined from experiments described in (**B**). Bars represent the mean ± SEM of this ratio per cell from three independent experiments. In (**C**), *** *p* < 0.001, determined by one-way ANOVA with Dunnett’s multiple comparisons test. In (**D**), *** *p* < 0.001, calculated using the unpaired Student’s *t* test.

To investigate whether the degradation defect of phagocytic vesicles induced by PRRSV is attributable to lysosomal damage, Protonex™ 600 Red-latex beads conjugate, which emit a red light under acidic conditions and lysosomes undergo acidification upon fusion with phagocytic vesicles, were introduced into cells subjected to various treatments. The results showed that a decline in lysosomal acidification with increasing viral load (Figure 4B,D). Concurrently, we examined the co-localization of PRV-pAb-FITC complexes with endosomes and observed that FITC fluorescence was not quenched in PRRSV-infected cells, further indicating that lysosomes may undergo a process rendering them incapable of acidification (Figure 4A,C). We speculate that PRRSV disrupts the integrity of the lysosomal membrane, leading to a reduction in luminal proton concentration and, consequently, the inability of lysosomes to acidify.

### 2.5. ROS Mediate PRRSV-Induced Lysosomal Damage and Phagocytic Defects

**Figure 5:**
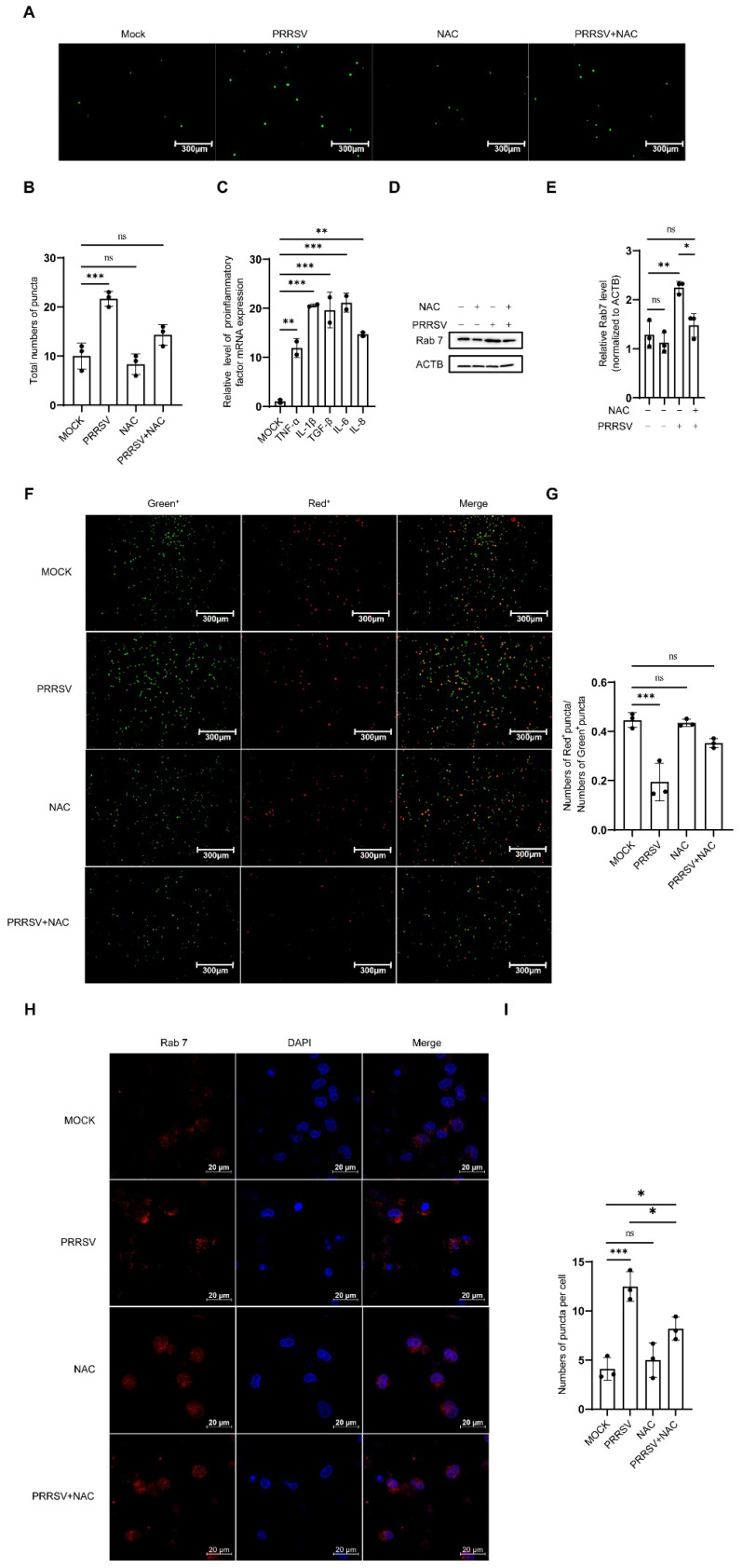
ROS mediate PRRSV-induced lysosomal damage and phagocytic defects. (**A**), fluorescence microscopy images of PAMs treated with 1 MOI of PRRSV and/or 1 mM NAC for 24 h, followed by loading with 10 μM DCFH-DA for 0.5 h. Scale bar: 300 μm. (**B**), the fluorescence intensity in each of the same fields of view was measured according to the experiments in (**A**). Bars represent the mean ± SEM of the fluorescence intensity per cell from three independent experiments. (**C**), mRNA levels of pro-inflammatory factors in PAMs infected with PRRSV for 24 h. (**D**), PAMs were treated with 1 MOI of PRRSV and/or 1 mM NAC for 24 h, followed by the addition of PRV-pAb complex for 7 h. Samples were then collected for immunoblotting. (**E**), quantification of Rab7 levels (normalized to ACTB) from experiments similar to those in (**D**). The ratio of Rab7: ACTB for the control group was arbitrarily set at 1. Bars represent the mean ± SEM from three independent experiments. (**F**), PAMs were treated with 1 MOI of PRRSV and/or 1 mM NAC for 24 h, after which Protonex^TM^ 600 Red-latex beads conjugate was added for 4 h. CytoTrace^TM^ Green working solution was then added for 0.5 h, and the cells were observed using a confocal microscope. Scale bar: 300 μm. (**G**), the ratio of the number of red-positive puncta to green-positive puncta within the same field of view was determined from experiments described in (**F**). Bars represent the mean ± SEM of this ratio per cell from three independent experiments. (**H**), confocal microscopy images of PAMs treated with 1 MOI of PRRSV and/or 1 mM NAC for 24 h, and then immunostained with antibodies against Rab7. Nuclei were stained with DAPI. Scale bar: 20 μm. (**I**), the fluorescence intensity in each of the same fields of view was measured according to the experiments in (**A**). Bars represent the mean ± SEM of the fluorescence intensity per cell from three independent experiments. In (**B**,**C**,**E**,**G**,**I**), * *p* < 0.05, ** *p* < 0.01, *** *p* < 0.001, determined by one-way ANOVA with Tukey’s multiple comparisons test.

PRRSV infection is accompanied by the generation of reactive oxygen species (ROS). Consequently, we hypothesized that ROS compromises the integrity of the lysosomal membrane, leading to defective phagocytic degradation. Initially, we measured the mRNA levels of several pro-inflammatory factors and found that PRRSV infection significantly upregulated their expression, and these factors induce ROS production (Figure 5C). We then observed intracellular ROS using the redox probe 2′,7′-dichlorofluorescein diacetate (DCFH-DA). As shown in Figure 5A,B, PRRSV-treated PAMs exhibited a high level of DCFH-DA fluorescence, which could be reduced by the commonly used ROS scavenger N-acetylcysteine (NAC). To investigate whether PRRSV-induced ROS regulate lysosomal stability, we measured lysosomal pH by treating PAMs differently, adding Protonex™ 600 Red-latex beads conjugate, and observing their luminescence. We observed that NAC significantly promoted lysosomal red-light fluorescence, which was inhibited by PRRSV, and that NAC increased the average fluorescence intensity per PRRSV-treated cells (Figure 5F,G). These observations suggest that PRRSV-induced lysosomal damage is attenuated by ROS depletion and that NAC rescues PRRSV-induced lysosomal acidification loss. Next, we further evaluated the role of reactive oxygen species in PRRSV-induced phagocytic defects. Immunoblot analysis revealed that NAC treatment reduced the accumulation of Rab7 by approximately 0.3-fold (Figure 5D,E). This finding is consistent with an immunofluorescence assay showing a reduction in Rab7 levels. Analysis of phagocytic flux using PRV-pAb complexes demonstrated that NAC alleviated the accumulation of endosomal positive structures (Figure 5H). Compared to PRRSV-treated cells, NAC treatment decreased the ratio of the number of red spots from approximately 12.50 to approximately 8.18 (Figure 5I). Taken together, these findings suggest that ROS are key mediators in PRRSV-induced lysosomal damage and phagocytic inhibition, and that these effects can be mitigated by NAC.

## 3. Discussion

PRRSV evades the host’s immune system through diverse mechanisms and modulates the antiviral immune response in a sophisticated and precise manner [41]. While establishing a persistent infection in the host, it significantly suppresses the host’s immune response, leading to secondary and mixed infections and causing substantial economic losses to the global pig farming industry. This also poses a formidable challenge for the prevention and control of PRRS [42]. Clinical and experimental studies have shown that PRV is a common secondary infection after PRRSV infection in the host. Other common secondary infections include Streptococcus suis (SS), Porcine respiratory coronavirus (PRCV), and Swine influenza (SI). A notable feature of these triggered secondary infectious diseases is that they all cause some degree of lung damage [19]. Literature reports suggest that experimental infection of piglets with PRRSV, when combined with SS, PRCV, or SI, accelerates the progression of these clinical diseases, although these pathogens do not directly target PAMs [25]. Our experiments, for the first time, revealed that lysosomal membrane damage induced by elevated ROS levels caused by PRRSV inhibits the degradation of PRV or PRV-antibody complexes. Lysosomes are pivotal organelles in macrophages, responsible for degrading pathogens, regulating immune responses, and maintaining tissue homeostasis [43]. Consequently, we hypothesize that the processes of PRV presentation and elimination by macrophages are disrupted, and that after inducing secondary infections, the inability to elicit an effective T-cell response may also contribute to the propagation of other viruses.

The physiological processes of phagocytosis and autophagy in macrophages are highly similar, both relying on the lysosomal degradation pathway and sharing the lysosomal degradation mechanism. Autophagy can indirectly support phagocytosis by removing damaged organelles (e.g., mitochondria), and the substances generated during phagosome formation and degradation can serve as substrates for autophagy [44,45,46,47,48]. In this study we found that NAC could partially alleviate the phagocytic flux but could not restore it to normal levels, suggesting that ROS-induced lysosomal damage may be only one of the reasons why PRRSV inhibits phagocytosis in PAMs. Our previous studies showed that PRRSV GP5 protein targets LAMP2A to inhibit chaperone-mediated autophagy (CMA) in macrophages, thereby facilitating the antiviral effect of PRRSV by escaping CMA [49]. Yanrong Zhou et al. also found that the PRRSV NSP5 protein induces incomplete autophagy by impairing the interaction of STX17 and SNAP29 [50]. Han Gu et al. showed that viral nonstructural proteins interfere with lysosomal homeostasis by hijacking the host cell MALT1 protein, preventing the autophagosome from completing its degradation function and transforming it into a carrier for viral replication and propagation [51,52]. Although these studies focused on the autophagy, they ultimately revealed that PRRSV inhibited the degradation of autophagosomes through different proteins that impair lysosomal capacity. Based on the similar lysosomal degradation mechanism of autophagy and phagocytosis, we hypothesize that PRRSV affects phagocytosis simultaneously with autophagy, and the above findings are likely the reasons why PRRSV affects phagosome degradation. However, more in-depth studies are needed to verify these hypotheses.

The process of phagocytosis in macrophages consists of several stages: detection of particles to be ingested, activation of the internalization process, formation of phagolysosomes, maturation of phagolysosomes, and their conversion into phagolytic lysosomes. This can be succinctly divided into three processes: internalization, maturation, and degradation [53]. After adding the PRV-antibody-FITC complex for incubation without allowing PAMs to digest it, we directly collected samples for observation. We found that the amount of phagocytosis of the complex in PRRSV-infected PAMs was significantly less than that in normal cells, suggesting that the internalization process of the complexes is also affected to some extent during PAM phagocytosis of immune complexes. Previous studies have demonstrated the existence of the Antibody-Dependent Enhancement (ADE) phenomenon of PRRSV, which plays a key role in the pathogenesis [54,55]. Based on this, Wanbo et al. found that both activating receptors FcγRI and FcγRIII were rapidly down-regulated in PRRSV-infected PAMs, whereas the inhibitory Fc receptor FcγRII were slightly up-regulated after infection. These changes contributed to promoting the ADE effect of PRRSV, resulting in a reduced total number of Fcγ receptors [56]. We hypothesize that this may be responsible for the reduced internalization of other immune complexes by PAMs. Similarly, Miet I De Baere1 et al., in studying the effect of PRRSV on phagocytosis in PAMs, demonstrated that the European genotype PRRSV inhibits in vitro PAM phagocytosis through interactions with its internalized receptor Sn [57]. Their study also interpreted the findings from the perspective of internalization, without detailed discussion of subsequent processes.

**Figure 6:**
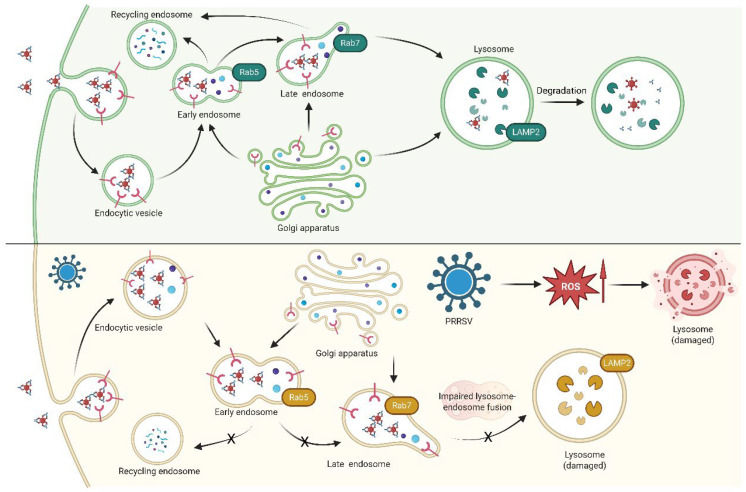
A model shows that PRRSV suppresses the phagocytic capacity of PAMs by inducing excessive ROS damage to lysosomes.

Our study is the first to elucidate how PRRSV affects the phagocytosis of PAMs from the perspective of degradation (Figure 6). We used PRV-antibody complexes as a standard for phagocytic flux and, for the first time, intuitively demonstrated that PRRSV infection interferes with the phagosome maturation process regulated by Rab proteins. Rab7 plays a key role in the fusion of phagosomes and lysosomes. After phagocytosis of pathogens by normal cells, the Rab7 content gradually decreases with phagosomal degradation. However, in PRRSV-infected cells, the Rab7 content did not change significantly within 7 h, implying that PRRSV obstructed the normal degradation of phagosomes. Meanwhile, PRRSV also affected the regulation of other Rab proteins (e.g., Rab5, Rab9, and Rab11). In normal cells, the processing, degradation, and intracellular recycling of pathogen complexes can be completed within 6 h; however, in PRRSV-infected cells, this entire process is impaired. Through further analysis of changes in the levels of various endosomal markers at specific time points, we found that Rab 7 levels in the infected group decreased around the 5 h mark and then rebounded at 7 h (Figure S1); whereas Rab 5, Rab 9, and Rab 11 also exhibited a brief decline in levels before 7 h, followed by a rebound. We speculate that this phenomenon may result from lysosomal overload caused by damage occurring around 5 h following PRRSV infection, leading to an accumulation of endosomes due to the inability to process phagocytosed material. However, the impact of PRRSV infection on PAM is complex and profound, and further in-depth research is required to elucidate the specific mechanisms (Figure S2). Further studies revealed that PRRSV inhibits phagosomal degradation by blocking phagosome-lysosome fusion. Experiments using the mTOR inhibitors rapamycin and chloroquine to treat cells confirmed that PRRSV does not elevate Rab7 levels by increasing phagocytic vesicle formation but rather blocks the process of phagocytic vesicle degradation. In normal cells, phagocytic vesicles fuse normally with lysosomes to form phagolysosomes, but this fusion is significantly reduced after PRRSV infection, directly affected the clearance efficiency of the pathogen. ROS play a crucial role in the process of PRRSV infection, upregulating the expression of pro-inflammatory factors and inducing ROS production. ROS levels are significantly elevated in PRRSV-treated PAMs, and the use of the ROS scavenger N-acetylcysteine (NAC) reduced ROS levels and alleviates PRRSV-induced lysosomal damage and phagocytosis defects (Figure 5). In conclusion, PRRSV infection is a complex process involving multiple cellular pathways and molecular interactions. Understanding the molecular mechanisms by which PRRSV inhibits the phagocytosis of PAMs will facilitate accurate prevention and control of PRRSV infections, provide a theoretical basis for the development of more effective preventive and control measures, and minimize the losses caused by PRRSV to the pig industry.

## 4. Materials and Methods

### 4.1. Reagents and Antibodies

RPMI 1640 Medium (90022), iFluor 594-Wheat Germ Agglutinin (WGA) Conjugate(W11262), Technology Co. 4′,6-diamidino-2-phenylindole (DAPI; C0065) and Bovine Serum Albumin (BSA; 9048-46-8) were purchased from Solarbio® Life Sciences (Beijing, China). The FITC Labeling Kit (E-LK-F003C) was obtained from Elabscience Biotechnology Co., Ltd. (Wuhan, China). The Rab7 Antibody (B-3): sc-376362 was purchased from Santa Cruz Biotechnology (Dallas, TX, USA). Rab5 Recombinant Rabbit Monoclonal Antibody (ET1609-27), Rab11A Recombinant Rabbit Monoclonal Antibody (HA721552), Rab9 Recombinant Rabbit Monoclonal Antibody (ET1602-29), HRP-conjugated Goat Anti-Swine IgG (SA00001-5), HRP-conjugated Goat Anti-Mouse IgG(HA1006), CD107b/LAMP2 Monoclonal antibody(HA601192), HRP-conjugated Goat Anti-Rabbit IgG (HA1001), and Beta Actin Monoclonal antibody(EM21002) were purchased from Hangzhou HuaAn Biotechnology Co., Ltd. (Hangzhou, China). Alexa Fluor^TM^ 546 goat anti-mouse IgG (A-11003) was purchased from Invitrogen Corporation (Waltham, MA, USA). Chloroquine (CQ; HY-17589A), Rapamycin (RAPA; HY-10219), and NAC(HY-B0215) were purchased from MedChemExpress LLC (Monmouth Junction, NJ, USA). The Cell Meter™ Fluorimetric Phagocytosis Assay Kit Red Fluorescence (Protonex 600 Red-Latex Beads Conjugate, CytoTrace™ Green; 21225) was purchased from AAT Bioquest, Inc. (Sunnyvale, CA, USA). RNA isolater Total RNA Extraction Reagent(R401-01), HiScript II Q RT SuperMix for qPCR (+gDNA wiper) (R233-01), and Taq Pro Universal SYBR qPCR Master Mix(Q712-02/03) were purchased from Nanjing Vazyme Bio-technology Co., Ltd. (Nanjing, China). Protein G Sepharose 4 Fast Flow (17061801) was purchased from Cytiva (Washington, MA, USA). DCFH-DA(S1105S) was purchased from Shanghai Beyotime Biotechnology Co. (Shanghai, China). Anti-PRRSV N protein antibody anti-PRRSV N protein antibody was stored in the laboratory.

### 4.2. Cell Culture and Viral

Porcine alveolar macrophages (PAMs) are typically obtained from the lungs of live pigs via bronchoalveolar lavage (BAL). Briefly, healthy, specific pathogen-free (SPF) pigs are fasted for a period before anesthesia and proper positioning. A catheter is inserted into the airway, and sterile physiological saline (20–50 mL per instillation, total volume up to 100–200 mL) is instilled into the lungs and then slowly aspirated. This lavage procedure is repeated several times to collect fluid enriched with alveolar macrophages. The collected lavage fluid is centrifuged to pellet the cells, and the supernatant is discarded. Erythrocytes are subsequently removed using a red blood cell lysis buffer. PAMs were cultured in a 5% CO_2_ incubator with RPMI 1640 Medium supplemented with 10% fetal bovine serum (FBS, Gibco; Grand Island, NY, USA) at 37 °C. PRRSV HN07-1 viruses and PRV viruses (ZJ-45 strain) with anti-PRV sera were maintained in our laboratory.

### 4.3. Viral Infection and the Use of PRV-pAb Complexes to Trigger Phagocytosis

PAMs were seeded in 12-well plates for 12 h and subsequently infected with different inoculum amounts of HN07-1 (MOI = 0.01, 0.1, 0.5, 1). After 24 h of infection, 200 μL of PRV complexed with anti-PRV antibody was added to each well (PRV-pAb complexes were pre-incubated for one hour, with the ratio of PRV-pAb complexes in the present study being PRV:pAb = 2000 TCID_50_:850 μg per milliliter of the mixture). PAMs were collected at different time points and analyzed by Western blotting (WB), flow cytometry, and indirect immunofluorescence assay (IFA).

### 4.4. Enzyme-Linked Immunosorbent Assay

The potency of anti-PRV serum was determined by indirect ELISA. Briefly, PRV antigen was added to an enzyme-labeled plate at 1 μg/mL in coating buffer (CBS, 0.05 M carbonate buffer, pH 9.6), 100 μL/well, and sealed with a laminating film to prevent water evaporation. The plate was incubated overnight (>12 h). The next day, the liquid was discarded, and the plate was dried on absorbent paper. Subsequently, 150 μL of blocking solution (CBS containing 2% non-fat milk) was added per well and incubated for more than 2 h at room temperature (25 °C). The plate was washed three times with PBST [PBS containing 0.05% Tween-20 (pH 7.4)]. Positive and blank sera were serially diluted starting from 1:500 with the antibody diluent (PBS buffer containing 1% non-fat milk, pH 7.4). After washing, 100 μL of secondary antibody working solution (HRP-labeled sheep anti-pig IgG diluted 1:3000 in antibody diluent) was added to each well and incubated at 37 for 1 h. Following washing, 100 μL of two-component TMB substrate (Frdbio, Cat No.: ELS0010; Hangzhou, China) was added to each well and incubated at room temperature for 0.25 h. Finally, 50 μL of termination solution (0.5 M H_2_SO_4_) was added to each well. The absorbance at 450 nm was read on a multifunctional enzyme labeler (Spark 10M, Tecan Trading AG, Männedorf, Switzerland).

### 4.5. Purification of Anti-PRV Antibody and Coupling FITC

The antibody was purified using Protein G Sepharose 4 Fast Flow. Briefly, 1 mL of Protein G packing was added to the equilibrium column and washed with 5 column volumes of 20% ethanol, followed by the addition of 5 column volumes of binding buffer (20 mM sodium phosphate, pH 7.0) at a controlled flow rate of 1 mL/min. 10 mL of serum (1:1 diluted with binding buffer) was added, and the sampling was repeated twice. The antibody was eluted using elution buffer (0.1 M glycine buffer, pH 3.0 to 2.5), and the eluted fraction was neutralized with buffer (1 M TrisHCl, pH 9.0). The final concentration was measured, and the antibody was stored with 20% glycerol. The antibody was filtered through a 0.45 μm membrane and used.

The anti-PRV antibody was coupled with fluorescein isothiocyanate (FITC) using FITC Labeling Kit. 1 mg of the antibody to be labeled was concentrated by ultrafiltration, and the total volume was adjusted to 0.5 mL with labeling buffer. Then, 13.3 μL of 25.7 mM FITC was added, gently mixed, and incubate in a constant temperature box at 37 °C for 0.5 h in the dark. The antibody was concentrated again by ultrafiltration to, remove the unbound FITC, and the solution was diluted with 1×PBS to 500 μL by centrifugation and ultrafiltration, repeated 2–3 times until the ultrafiltrate in the collection tube was nearly colorless and transparent. The FITC-labeled anti-PRV antibody was obtained by adding 1×PBS to a final volume of 500 μL.

### 4.6. Western Blotting

PAMs were collected and lysed with RIPA lysis buffer. Protein samples were separated by 12% SDS-PAGE and transferred to the polyvinylidene fluoride (PVDF) membranes (C3117, Millipore, MA, USA). The membrane was blocked with 5% non-fat milk (A600669, Sangon Biotech, Shanghai, China) for 2 h. The membrane was then incubated with the primary antibody overnight at 4 °C and with the secondary antibody at room temperature for 2 h. The membrane was washed three times with PBST after each step. The results were analyzed using the SuperPico ECL Chemiluminescence Kit (Vazyme Biotech Co., Ltd, Cat No.: E22; Nanjing China). Anti-Rab7 antibody, anti-Rab5 antibody, anti-Rab9 antibody, and anti-Rab11 antibody were used to detect Rab7, Rab5, Rab9, and Rab11 proteins, respectively; β-actin rabbit antibody was used to stain β-actin as a loading control. HRP-coupled affinity goat anti-rabbit IgG (H+L) or HRP-coupled affinity goat anti-mouse IgG (H+L) was used as secondary antibody.

### 4.7. Indirect Immunofluorescence Assay (IFA)

Cells grown on coverslips were washed with phosphate-buffered saline (PBS) and treated PAMs were fixed with 4% paraformaldehyde for 0.25 hat room temperature and immediately permeabilized with pre-cooled Triton X-100 for 10 min. After blocking with 5% BSA, the cells were incubated with the indicated antibodies for 1 h at room temperature. After thorough washing, cells were treated with Alexa Fluor546-conjugated anti-mouse antibody or FITC-conjugated goat anti-mouse IgG for 1 h. After washing, DAPI was added to stain the cells at room temperature for 10 min. Fluorescence images were obtained by confocal microscopy (LSM800, CarlZeiss, Germany). To observe intracellular phagocytosis of coupled FITC anti-PRV antibody-PRV complexes, cell membranes were stained using iFluor 594 wheat germ agglutinin (WGA) affix.

### 4.8. Lysosomal Acidification Assay

12.5 μL Protonex 600 Red Latex Beads Conjugate solution was added to each well of PAMs in a 24-well plate. The dishes were incubated in a cell incubator for 4 h. Then, 12.5 μL of cyto-trace green working solution was added to each well to indicate cell activity, followed by further incubation in a cell culture incubator for 0.5 h. After incubation, the cells were washed twice with PBS. Lysosomal acidification within the cells was observed using a Texas red filter (Ex/Em = 570/600 nm) and a FITC green filter (Ex/Em = 490/525 nm).

### 4.9. Flow CytoMetry (FCM)

PAMs incubated with coupled FITC-PRV complexes were collected into 1.5 mL EP tubes, washed three times with PBS, and transferred to flow tubes (Franklin Lakes, NJ, USA) for analysis using a CytoFLEX flow cytometer (Beckman Coulter, Brea, CA, USA).

### 4.10. Quantitative Real-Time PCR (qRT-PCR) Assay

To determine the levels of changes in associated M1-type pro-inflammatory factors, viral RNA was extracted from PRRSV-infected PAMs using RNA isolater Total RNA Extraction Reagent. RNA was reverse transcribed into cDNA according to the instructions of the HiScript II Q RT SuperMix for qPCR kit. cDNA was then amplified in an ABI 7500 real-time PCR system (Applied Biosystems; Waltham, MA, USA) using Taq Pro Universal SYBR qPCR Master Mix. The primers for qRT-PCR (qRT-PCR-F/qRT-PCR-R) are listed in Table 1.

### 4.11. Statistical Analysis

For quantitative analyses, data were acquired from at least three independent experiments and presented as means ± Standard Deviation (SD). The significance levels were predetermined at 0.05, 0.01, and 0.001, denoted by *, **, and ***, respectively. Statistical analysis was performed using GraphPad Prism 8 (GraphPad Software, Inc, Boston, MA, USA) and ImageJ software (v 1.4.3.67). Subsequently, flow cytometry data were then analyzed with FlowJo software (v10.8.1). When comparing the mean differences between two independent sample groups, the unpaired Student’s *t*-test is employed to determine whether a significant difference exists between the two independent groups. For comparisons involving one control group versus multiple experimental groups, one-way ANOVA followed by Dunnett’s test is used to evaluate whether each treatment group differs from the same control group, with no focus on comparisons between experimental groups themselves. When conducting all possible pairwise comparisons among three or more groups, one-way ANOVA with Tukey’s multiple comparison test is applied. This approach comprehensively compares differences between every pair of groups while controlling the overall Type I error rate.

## 5. Conclusions

In conclusion, by employing PRV-antibody complexes as a marker for phagocytic flux, we have, for the first time, visualized the impact of PRRSV on the phagocytosis of PAMs. Our findings indicated that lysosomal membrane damage, resulting from PRRSV-induced elevation of ROS levels, inhibits the degradation of PRV or PRV-antibody complexes, thereby blocking phagosomal degradation. ROS play a pivotal role in this process, offering a novel perspective for comprehending the phenomena of PRRSV-induced immunosuppression and secondary infection.

## Figures and Tables

**Table 1:** qRT-PCR primers.

Cytokine	qRT-PCR-F	qRT-PCR-R
IL-1β	ATGCTGAAGGCTCTCCACCTC	TTGTTGCTATCATCTCCTTGCAC
IL-6	AAGCGCCTTCAGTCCAGTC	CCGGAGAGGTGAAGAGCATT
IL-8	TACGCATTCCACACCTTTCCA	ACAACCTTCTGCACCCACTTT
TNF-α	TGGTGGTGCCGACAGATGG	GGCTGATGGTGTGAGTGAGGAA
TGF-β	CTTACTGAGCATCTTGGACCTTA	CCACTGAGCCACAATGGAAA

## Data Availability

The original contributions presented in this study are included in the article/Supplementary Material. Further inquiries can be directed to the corresponding authors.

## Supplementary Materials

The following supporting information can be downloaded at: https://www.mdpi.com/article/10.3390/ijms27135800/s1.
