# Supplementary material for: Porcine Reproductive and Respiratory Syndrome Virus (PRRSV)-Induced Reactive Oxygen Species Inhibit Phagocytosis in Alveolar Macrophages
Source: Int J Mol Sci. 2026 Jun 26;27(13):5800. doi: 10.3390/ijms27135800 (PMC13361384; doi:10.3390/ijms27135800)
Supplement: Supplementary file 1 [file ijms-27-05800-s001.zip › ijms-4177409-supplementary.pdf]

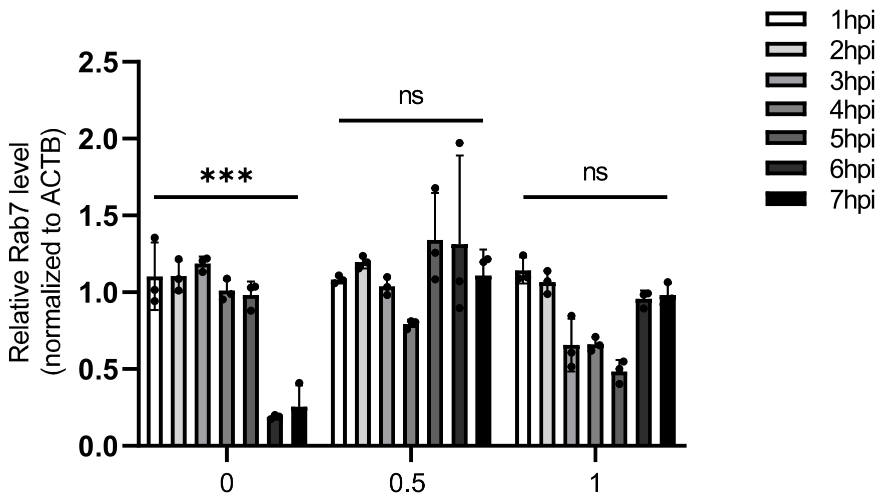

Supplementary Figure S1. Quantitative analysis of Rab7 levels at all time points, normalized to ACTB, derived from experiments conducted as described in Figure 2C. The ratio of each indicated protein to ACTB for the control group was arbitrarily set at 1. Bars represent the mean ± SEM from three independent experiments. In B, statistical significance was determined using one-way ANOVA with Tukey's multiple comparisons test (*p < 0.05, **p < 0.01, ***p < 0. 001).

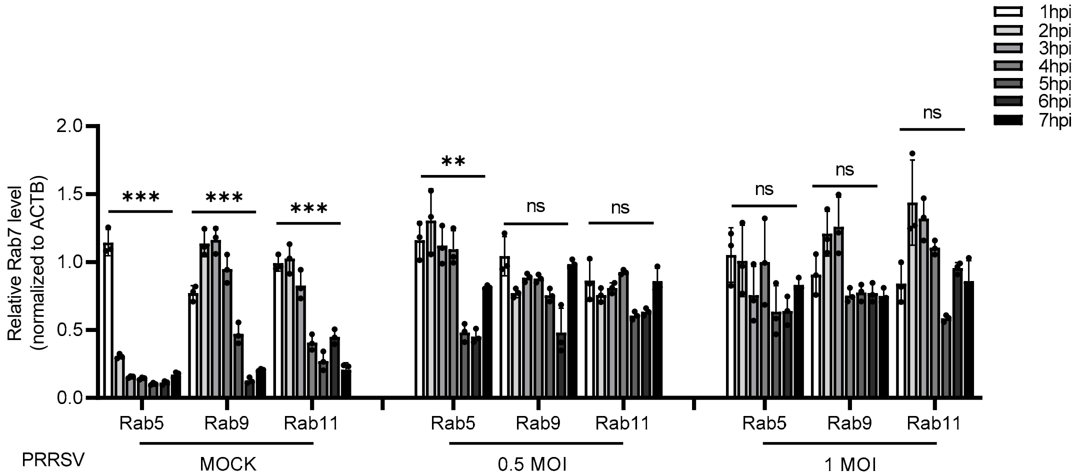

Supplementary Figure S2. Quantitative analysis of Rab5, Rab9, and Rab11 levels at all time points, normalized to ACTB, derived from experiments conducted as described in Figure 3A. The ratio of each indicated protein to ACTB for the control group was arbitrarily set at 1. Bars represent the mean ± SEM from three independent experiments. In B, statistical significance was determined using one-way ANOVA with Tukey's multiple comparisons test (*p < 0.05, **p < 0.01, ***p < 0. 001).
